# Screening of circRNAs Associated with Secondary Wool Follicle Development in Fine-Wool Sheep and Construction of Their ceRNA Network

**DOI:** 10.3390/ani15172629

**Published:** 2025-09-08

**Authors:** Yu Luo, Binpeng Xi, Yufang Song, Tong Xiao, Zengkui Lu, Jianbin Liu, Chao Yuan, Tingting Guo

**Affiliations:** 1 Key Laboratory of Animal Genetics and Breeding on the Tibetan Plateau, Ministry of Agriculture and Rural Affairs, Lanzhou Institute of Husbandry and Pharmaceutical Sciences, Chinese Academy of Agricultural Sciences, Lanzhou 730050, China; 17393144478@163.com (Y.L.); w5562080w@163.com (B.X.); songyf805@163.com (Y.S.); xiaotong3110134994@163.com (T.X.); luzengkui@caas.cn (Z.L.); liujianbin@caas.cn (J.L.); yuanchao@caas.cn (C.Y.); 2 Sheep Breeding Engineering Technology Research Center of Chinese Academy of Agricultural Sciences, Lanzhou 730050, China

**Keywords:** fine-wool sheep, secondary wool follicle, development, circRNA, circRNA–miRNA–mRNA

## Abstract

Various signalling molecules and pathways critical for wool production and quality regulate wool secondary follicle (SF) development. Circular RNAs (circRNAs) regulate SF morphogenesis through a competing endogenous RNA (ceRNA) mechanism; these novel cyclic non-coding RNAs are not known to regulate the development of SFs in the foetal period of fine-wool sheep. Here, we analysed circRNA expression profiles in the foetal skin of Gansu Alpine fine-wool sheep at 10 developmental stages (E87, E90, E93, E96, E99, E102, E105, E108, E111, and E138) using RNA sequencing. Among the differentially expressed circRNAs (DE circRNAs), 173 were significantly enriched in signalling pathways related to hair follicle (HF) development, such as Wnt/β-catenin, transforming growth factor-β/Smad, Notch, and mitogen-activated protein kinase. Six HF-development-related circRNAs were expressed at different stages and potentially regulated SF development through the ceRNA network. In total, 16 DE circRNAs and their targets, 44 miRNAs, and 65 mRNAs were screened, and 88 ceRNA regulatory network pathways related to SF development were constructed. These key DE circRNAs could be candidate genes for further exploration of the molecular HF development mechanism, providing an important theoretical basis for unravelling the regulatory network of SF development in fine-wool sheep and genetic wool trait improvement.

## Simple Summary

Wool is an important raw material of animal origin that is irreplaceable in national economies. Improving the yield of high-quality fine-wool sheep has been the core goal of fine-wool-sheep-breeding research. Wool growth is a complex physiological and biochemical process regulated by a variety of factors. Secondary follicle (SF) development directly determines wool yield and quality. In this study, we investigated the role of circular RNA (circRNA) in regulating SF development at ten key embryonic stages in Alpine Merino sheep. The expression profiles of circRNAs were constructed by RNA sequencing, and 173 differentially expressed circRNAs were found to be significantly enriched in core pathways of hair follicle development, such as Wnt/β-catenin, transforming growth factor-β/Smad, Notch, and mitogen-activated protein kinase. Further construction of 88 circRNA–miRNA–mRNA regulatory networks showed that 16 key circRNAs interacted with 44 miRNAs and 65 mRNAs through a competing endogenous RNA mechanism. Among these, six candidate molecules, including circRNA_07722 and circRNA_08249, provided important targets for analysing the molecular regulatory network of autologous fat development. These findings not only deepen our understanding of the regulatory mechanism of wool growth but also provide a new theoretical basis and technical route for the molecular breeding of fine-wool sheep.

## 1. Introduction

Wool, an important animal-derived textile raw material, plays a significant role in national economies. Increasing the yield of high-quality fine wool is an important direction in the current research on fine-wool-sheep breeding. Wool growth is a complex physiological and biochemical process influenced by multiple factors, such as genetics, the environment, and nutrition. The hair follicle (HF) is the central structure for wool growth and, although small, has a complex structure consisting of a population of cells from the ectoderm and mesoderm [1]. HFs are mainly composed of the hair bulb (HB), the inner root sheath (IRS), the hair shaft (HS), the outer root sheath (ORS), the connective tissue sheath (CTS), etc. [2]. HFs are divided into primary HFs (PFs) and secondary HFs (SFs), of which PFs develop earlier and SFs later [3,4]. The growth and development status of SFs directly impact the yield and quality of wool [5]. The redifferentiation process is a key factor in regulating the density of wool follicles, which, in turn, effectively improves its fineness. HF morphogenesis involves the synergistic effects of a series of signalling pathways between the epidermis and dermis. HF development is regulated by multiple signalling pathways, including the Wnt, sonic hedgehog (SHH) conduction, Notch conduction, transforming growth factor (TGF) family, bone morphogenetic protein (BMP), and fibroblast growth factor (FGF) pathways [6,7,8,9,10,11].

Previous studies focused primarily on the functional level of genes. However, in recent years, the role of non-coding RNAs (ncRNAs) (such as long circular RNAs (circRNAs), non-coding RNAs (ncRNAs), and microRNAs (miRNAs)) at different stages of HF development has attracted considerable attention. Numerous studies have shown that ncRNAs are important post-transcriptional regulators of gene expression during HF development. circRNAs are novel endogenous ncRNA molecules widely present in mammalian cells, formed by the reverse-splicing of the 3′ and 5′ ends of mRNA precursors to create single-stranded closed circular structures. CircRNAs can be classified into four types based on their source and composition: intron circRNAs (ciRNAs), exon circRNAs (ecircRNAs), exon–intron circRNAs (elciRNAs), and intergene circRNAs (icircRNAs) [12]. Unlike linear RNA, circRNA, due to the lack of a 5′ cap structure and 3′ poly(A) tail in its circular structure, can resist exonuclease degradation and exhibit high stability and conservation [12,13]. CircRNAs are rich in miRNA binding sites and can act as miRNA molecular sponges to regulate the expression of host genes [14,15], interact with RNA-binding proteins [16], participate in protein translation [17], or act as competitive endogenous RNAs (ceRNAs) to regulate gene expression [18,19]. CircRNAs that regulate gene expression and participate in the regulation of life activities through competitive binding of miRNAs have become a research hotspot in related fields. ceRNA molecules competitively bind to the same miRNA through miRNA response elements (MREs), thereby regulating their expression [20]. Recent studies have shown that circRNAs are involved in the regulation of HF growth via a ceRNA mechanism. For example, circFTO and circCSPP1 competitively bind to miR-148a and miR-10a, enhancing the expression of *BMP7* to promote the proliferation of lakeal papillae cells [21]; circRNA-1967 upregulates the expression of *LEF1* by adsorbing miR-93-3p and regulates the proliferation and differentiation of HF stem cells in cashmere goats [22]. However, studies on the regulatory mechanism of circRNAs during embryonic wool follicle development in fine-wool sheep remain relatively scarce.

The development of wool follicles in fine-wool sheep shows clear temporal nodes and phase characteristics. Specifically, when the gestational age reaches 87 days, SFs begin to occur, and at 102 days, the primary SFs are redifferentiated at the cervical protrusion site to form SFs. At a gestational age of 108 days, a large number of redifferentiation phenomena occur in primary SFs. At a gestational age of 138 days, most primary wool follicles and some SFs have matured [23]. Therefore, based on these previous findings, in this study, ten foetal skin tissue samples from fine-wool sheep with gestational ages of E87, E90, E93, E96, E99, E102, E105, E108, E111, and E138 were selected, and RNA sequencing (RNA-seq) technology combined with bioinformatics analysis was used to resolve the expression profiles of circRNAs at different developmental stages, identify DE circRNAs, reveal the biological functions and signalling pathway characteristics, and construct a circRNA–miRNA–mRNA regulatory network. The ultimate objective was to provide a new perspective for clarifying the functional mechanism of circRNAs in wool follicle development in fine-wool sheep.

## 2. Materials and Methods

### 2.1. Moral Declaration

All animals were handled in strict accordance with the Animal Ethics Procedures and Guidelines of the People’s Republic of China. This study was approved by the Animal Ethics Committee of the Lanzhou Institute of Animal Husbandry and Veterinary Medicine, Chinese Academy of Agricultural Sciences (licence no.: SYXK-2019-010).

### 2.2. Sample Collection and Animal Processing

All fine-wool sheep used in this experiment were obtained from the Gansu Province Sheep Breeding Technology Extension Station (Huangcheng Town, Sunan County, Zhangye, China). Thirty pregnant ewes aged 2–3 years old with the same feeding conditions were selected (average fibre diameter of 19.0–21.5 μm), and these ewes were artificially inseminated with the sperm of rams of the same strain (wool fibre diameter of 19.20 μm). The day of fertilisation was designated as the 0th day of embryo development. Thirty foetuses were delivered by caesarean section from thirty different female animals at ten stages (E87, E90, E93, E96, E99, E102, E108, E111, and E138) of embryonic development, with three replicates at each time point. A section of the skin sample, collected 10 cm behind the foetal scapula, was obtained using skin biopsy punches (10 mm) (Biosharp, rxz509, Hefei, China). The skin samples were rinsed with phosphate-buffered saline, quickly loaded into freezing tubes before freezing in a liquid-nitrogen tank, and stored at −80 °C for total RNA extraction and used for transcriptome sequencing and qPCR analysis.

### 2.3. circRNA Sequencing

#### 2.3.1. Extraction of Total RNA from the Skin and RNA Inversion

Total RNA was extracted from the skin tissue of 30 fine-wool sheep foetuses by sampling 50–100 mg of sheep skin in liquid nitrogen using TRIzol reagent (Takara, Shiga, Japan) according to the manufacturer’s instructions. A NanoPhotometer^®^ spectrophotometer (IMPLEN, Palo Alto, CA, USA) and an Agilent 2100 Bioanalyzer (Agilent Technologies, Palo Alto, CA, USA) were used to detect the concentration and quality of extracted total RNA. The datasets used in the experiments were derived from high-throughput sequencing performed by Ouyi Biologicals (Shanghai, China) under Project No. HT2020-16026.

#### 2.3.2. Library Preparation and Sequencing

The extracted total RNA samples were prepared to build a library using TruSeq Stranded Total RNA with the Ribo-Zero Gold Kit (Illumina, San Diego, CA, USA), in accordance with the manufacturer’s instructions. The constructed RNA library was inspected and qualified using an Agilent 2100 Bioanalyzer (Agilent Technologies, Palo Alto, CA, USA) and sequenced using an Illumina sequencer.

#### 2.3.3. Identification and Characteristic Analysis of circRNA

After obtaining a large amount of double-ended sequencing data, the whole transcription library was constructed using Trimmomatic software (v0.39) [24] to remove adapters and low-quality base reads and obtain high-quality data. Subsequently, Hisat2 (v2.2.1.0) [25] was used to align the clean reads with the reference genome Oar_v4.0 to obtain the position and sample-specific sequence information, and the RscqQC suite (v2.6.4) was used to statistically analyse the proportions of alignment types and evaluate the results. After obtaining the BAM file, the reads of the genes were assembled and aligned using StringTie software (v1.3.3b) [26], and the transcripts of each sample were fused and spliced into a complete transcript. Based on the structural characteristics of circRNAs and the characteristics of splicing sequences, circRNAs were predicted using CIRI [27] software (v2.0.3), classified, and annotated, and the chromosome distribution and length were statistically analysed.

### 2.4. DE circRNA Screening and Functional Enrichment Analysis of Source Genes

After standardising the counts of each sample circRNA using DESeq software (v1.18.0) [28], differential expression analysis of circRNAs was performed using the edgeR package (v3.12.1) [29]. Differences between comparison groups (E90 vs. E87, E93 vs. E87, E96 vs. E87, E99 vs. E87, E102 vs. E87, E105 vs. E87, E108 vs. E87, E111 vs. E87, and E138 vs. E87) and the analysis involved three main steps: ① normalisation of the read count values of circRNAs; ② calculation of the hypothesis test probability (*p*-value) under the model; and ③ multiple hypothesis testing correction to obtain the false discovery rate (FDR) < 0.05. Based on the differential analysis results, the circRNAs exhibiting *p* < 0.05 and |log2FC| > 1 were chosen as DE circRNAs. The source genes of the DE circRNAs were analysed using Gene Ontology (GO, https://www.geneontology.org/, Date of visit: 3 July 2025) and the Kyoto Encyclopedia of Genes and Genomes (KEGG, http://www.genome.jp/kegg/, Date of visit: 5 July 2025).

### 2.5. Validation of DE circRNAs

To validate the RNA-seq data, nine DE circRNAs were randomly selected for the quantitative fluorescence assay by quantitative real-time polymerase chain reaction (qRT-PCR), using β-actin as the internal reference gene. All primers were designed and synthesised by Shenggong Bioengineering Co., Ltd. (Shanghai, China) (Table 1). DE circRNAs were amplified by PCR using the cDNA of Alpine Merino sheep embryonic skin as a template and detected by Sanger sequencing. The amplified product sequences were compared with the circRNA sequences obtained by RNA-Seq using MEGA 7.0. Then, qRT-PCR was performed using the reagent SYBR Premix Ex Taq II (Takara, Japan) on the Roche LightCycler^®^ 96 real-time fluorescence quantitative PCR instrument with the following program: 95 °C for 30 s, 40 cycles at 95 °C for 5 s, and 60 °C for 30 s. *β*-Actin was used as the reference gene, and the equivalent expression level was calculated via the 2^−∆∆CT^ method [30].

### 2.6. Construction of circRNA-miRNA-mRNA Interaction Network

CircRNA-targeted miRNAs were predicted using the miRanda software (v3.3a) and TargetScan (v7.0) [31]. miRTarBase (v6.1) was used to predict the mRNA interactions between circRNAs and miRNAs [32]. The circRNA–miRNA–mRNA interaction network was constructed using the Cytoscape software (v3.10.3) [33].

### 2.7. Statistical Analysis—Quantification of Gene Expression and Analysis

Statistical analysis was performed using SPSS software (version 22.0; SPSS Inc., Chicago, IL, USA), and graphs were plotted using Origin 2021 (OriginLab Corp., Northampton, MA, USA). Data were expressed as the mean ± standard error (SEM), and the RT-PCR results were analysed for relative quantification by the 2^−ΔΔCt^ method. Differences between groups were compared using the independent-samples *t*-test or one-way ANOVA, and the significance levels were set at *p* < 0.05 and <0.01 for significant and highly significant differences, respectively.

Statistical analysis was performed using SPSS software (version 22.0; SPSS Inc., Chicago, IL, USA), and graphs were plotted using Origin 2021 (OriginLab Corp., Northampton, MA, USA). First, the normality of the data distribution in each treatment group was evaluated using the Shapiro–Wilk test. For the independent-samples *t*-test, the F-test was applied to check the homogeneity of variance between the two groups before comparison. When conducting one-way ANOVA, Levene’s test was used to assess the equality of variances across multiple groups. Data were expressed as the mean ± standard error (SEM), and the RT-PCR results were analysed for relative quantification by the 2^−ΔΔCt^ method. Differences between groups were compared using the independent-samples *t*-test or one-way ANOVA, and the significance levels were set at *p* < 0.05 and < 0.01 for significant and highly significant differences, respectively.

## 3. Results

### 3.1. High-Throughput Sequencing Quality Assessment

**Figure 1:**
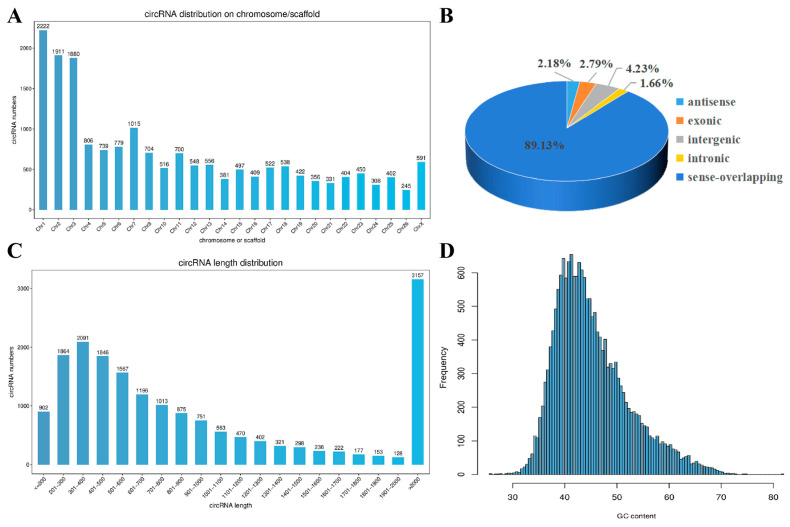
Analysis of the expression characteristics of circRNA in the skin tissue of alpine fine-wool sheep. (**A**) Distribution of circRNA numbers on each chromosome. (**B**) Classification of circRNAs in the wool follicles of fine-wool sheep. (**C**) Distribution map of circRNA sequence lengths. (**D**) Distribution map of circRNA GC contents.

To explore the expression pattern of circRNAs in the skin HFs of alpine fine-wool sheep during the developmental period, high-throughput sequencing was performed on skin tissue samples from fine-wool sheep foetuses at 10 different developmental stages (E87, E90, E93, E96, E99, E102, E105, E108, E111, and E138). Thirty RNA libraries of deribosomal HF development at different stages were constructed. After RNA sequencing on the Illumina HiSeq 4000 platform, 3,050,120,000 original reads were obtained. After quality control, 2,960,630,000 clean reads were obtained. The Q30 values of all of the samples were >98.10%. The GC content ranged from 45.90 to 49.44%. After removing ribosomal RNA, 94.03–95.53% of the clean reads were compared with the reference genome of fine-wool sheep. The overall assessment of the sequencing data is presented in Table S1. These clean data met both the quantity and quality requirements for subsequent circRNA analysis. In this study, 18232 circRNAs were identified, and their source genes were analysed. Novel circRNAs were categorised into five types: antisense (*n* = 398), exonic (*n* = 509), intergenic (*n* = 772), intronic (*n* = 303), and sense-overlapping (*n* = 16,250) circRNAs. The chromosome distribution showed that circRNAs were distributed on almost all chromosomes, with chromosomes 1–3 being the most enriched, and chromosomes 24 and 26 the least (Figure 1A). CircRNA types were predominantly sense-overlapping (89.13%), followed by intergenic (4.23%), exonic (2.79%), antisense (2.18%), and intronic (1.66%) in decreasing order (Figure 1B). Length-wise, the highest number of circRNAs were over 2000 bp (3157), followed by 301–400 bp (2091) (Figure 1C). The GC content of the circRNA sequences was in the range of 35–50% (Figure 1D).

### 3.2. circRNA Identification and Expression Analysis

**Figure 2:**
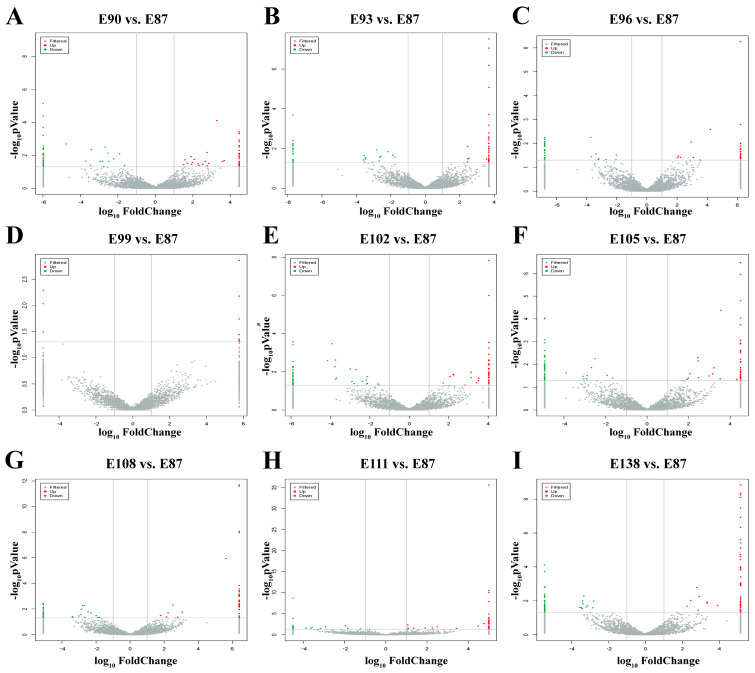
Volcano map of differentially expressed circRNAs. Grey represents circRNAs with non-significant differences, while red and green represent circRNAs with significant differences. (**A**) E90 vs. E87. (**B**) E93 vs. E87. (**C**) E96 vs. E87. (**D**) E99 vs. E87. (**E**) E102 vs. E87. (**F**) E105 vs. E87. (**G**) E108 vs. E87. (**H**) E111 vs. E87. (**I**) E138 vs. E87.

Using *p*-value < 0.05 and |log_2_FC| ≥ 1 as screening conditions, a total of 173 DE circRNAs were identified. Differentially expressed circRNAs were screened in the comparison groups (E90 vs. E87, E93 vs. E87, E96 vs. E87, E99 vs. E87, E102 vs. E87, E105 vs. E87, E108 vs. E87, E111 vs. E87, and E138 vs. E87) (Table S2). The results showed that, in the E90 vs. E87, E93 vs. E87, E96 vs. E87, E99 vs. E87, E102 vs. E87, E105 vs. E87, E108 vs. E87, E111 vs. E87, and E138 vs. E87 groups, 18, 6, 7, 0, 11, 13, 7, 10, and 8 were upregulated, whereas 13, 13, 8, 0, 17, 10, 13, 8, and 10 were downregulated (Figure 2). This lays the foundation for the subsequent screening of circRNAs related to SF development in fine-wool sheep.

### 3.3. Functional Enrichment Analysis of Target Genes

**Figure 3:**
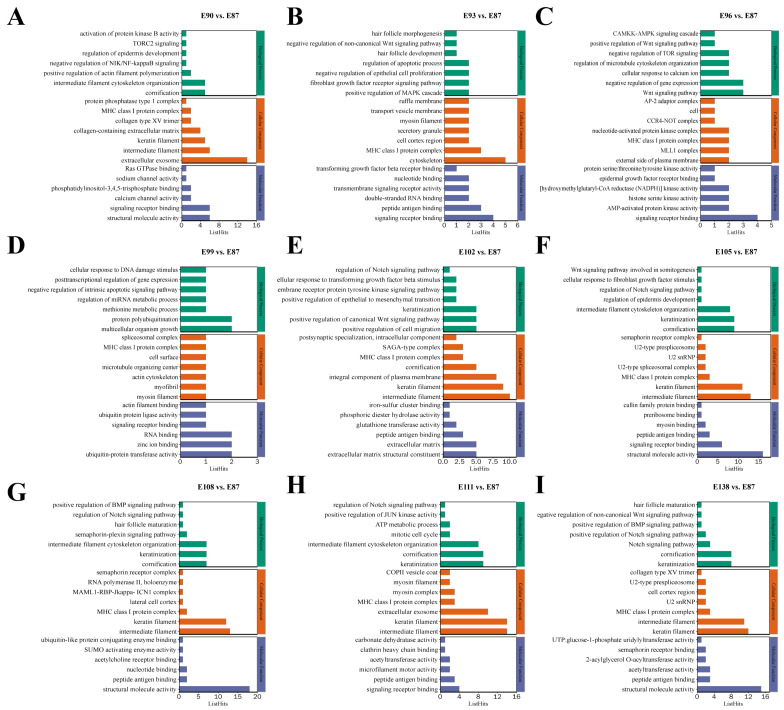
Functional annotation analysis of GO source genes. (**A**) E90 vs. E87. (**B**) E93 vs. E87. (**C**) E96 vs. E87. (**D**) E99 vs. E87. (**E**) E102 vs. E87. (**F**) E105 vs. E87. (**G**) E108 vs. E87. (**H**) E111 vs. E87. (**I**) E138 vs. E87.

**Figure 4:**
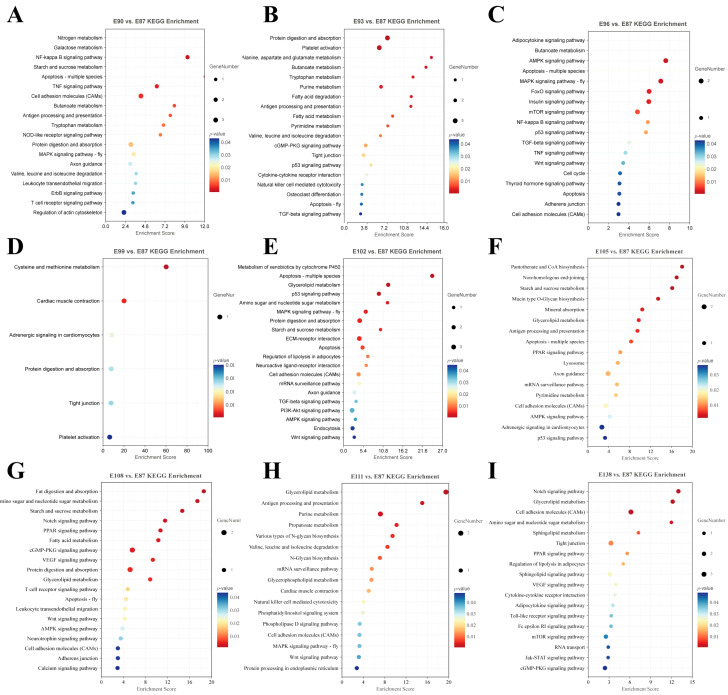
Analysis of the KEGG pathways of DE circRNAs’ target genes in each comparison group. (**A**) E90 vs. E87. (**B**) E93 vs. E87. (**C**) E96 vs. E87. (**D**) E99 vs. E87. (**E**) E102 vs. E87. (**F**) E105 vs. E87. (**G**) E108 vs. E87. (**H**) E111 vs. E87. (**I**) E138 vs. E87.

To explore the regulatory role of the target genes of the DE circRNAs in SF development in fine-wool sheep, GO and KEGG pathway enrichment analyses were performed (Tables S3 and S4). The results of GO functional annotation showed that the regulation of epidermis development (GO:0045682), HF development (GO:0001942), HF morphogenesis (GO:0031069), HF maturation (GO:0048820), Wnt signalling pathway (GO:0016055), skin morphogenesis (GO:0043589), SMAD protein signal transduction (GO:0060395), skin development (GO:0043588), regulation of Notch signalling pathway (GO:0008593), activation of MAPK activity (GO:0000187), keratin filament (GO:0045095), and epidermal growth factor receptor (GO:0005154) were significantly enriched (Figure 3). We used the KEGG database to analyse the transcriptome results and identified eight enriched pathways associated with secondary HF development: the Wnt (ko04310), TGF-β (ko04350), Notch (ko04330), NF-κB (ko04064), PI3K-Akt (ko04151), P53 (ko04115), and AMPK (ko04152) and mTOR (ko04150) signalling pathways (Figure 4).

### 3.4. Validation of the RNA-Seq Data

**Figure 5:**
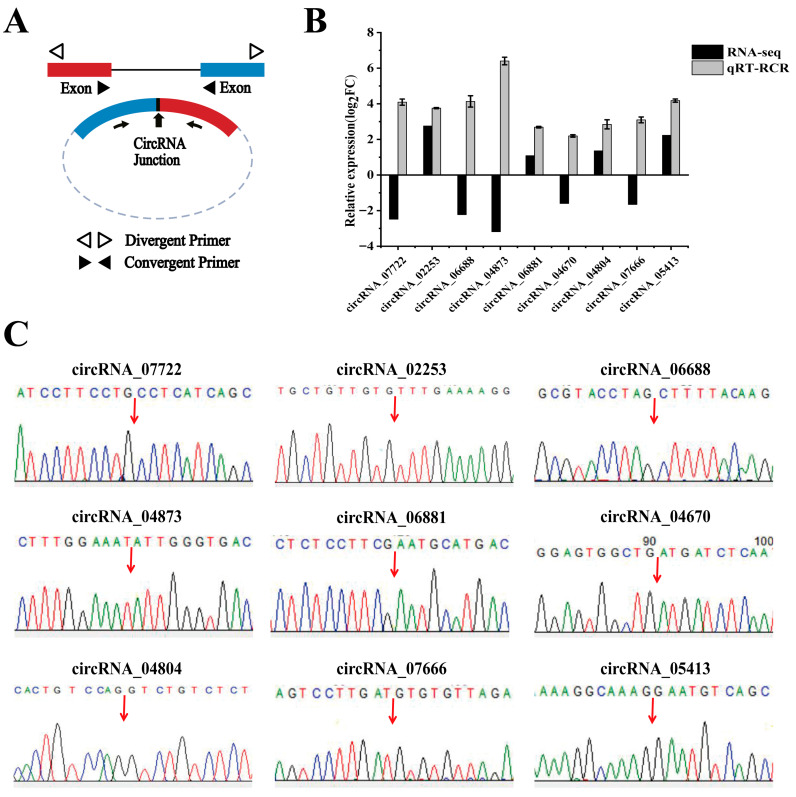
Verification of DE circRNAs from RNA-seq. (**A**) Schematic design of primers for DE circRNAs in qRT-PCR. (**B**) Comparison of 9 DE circRNA expression levels between RNA-seq and qRT-PCR. (**C**) Sanger sequencing of 9 DE circRNAs. *β*-Actin was used as the internal reference gene. Three replicates were set for qRT-PCR, and the results are expressed as the mean ± standard error (±SE). The cyclic site sequence of circRNAs in circbase are as follows: circRNA_07722 (ATCCTTCCTGCCTCATCAGC); circRNA_02253 (TGCTGTTGTGTTTGAAAAGG); circRNA_06688 (GCGTACCTAGCTTTTACAAG); circRNA_04873 (CTTTGGAAATATTGGGTGAC); circRNA_06881 (CTCTCCTTCGAATGCATGAC); circRNA_04670 (GGAGTGGCTGATGATCTCAA); circRNA_04804 (CACTGTCCAGGTCTGTCTCT); circRNA_07666 (AGTCCTTGATGTGTGTTAGA); and circRNA_05413 (AAAGGCAAAGGAATGTCAGC).

**Figure 6:**
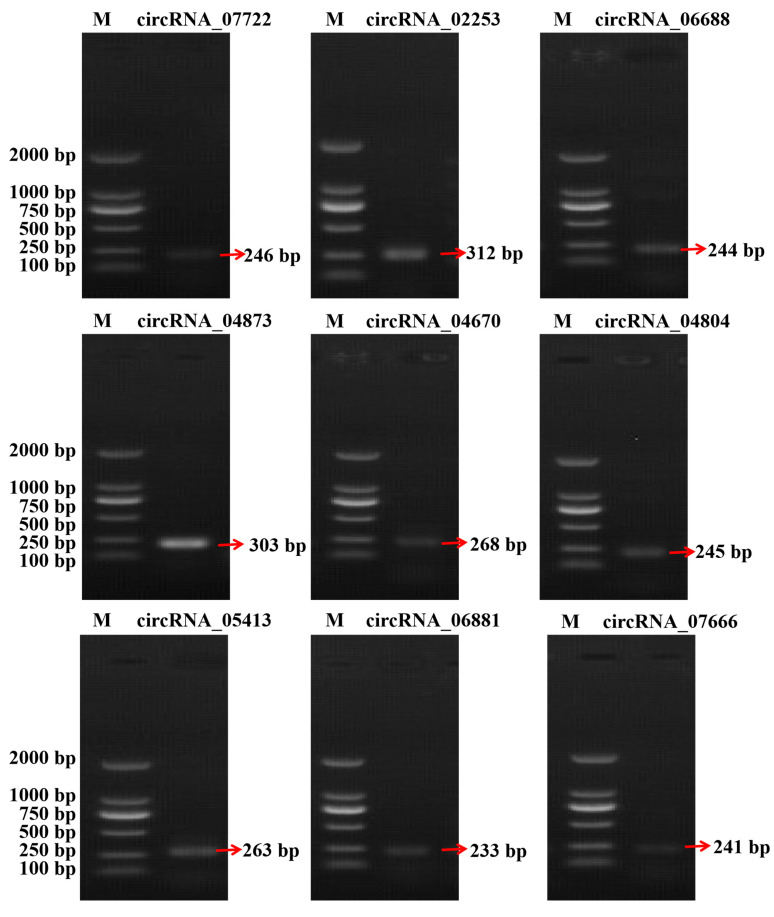
Agarose gel electrophoresis image of circRNAs amplified by PCR. Amplification bands of circRNA_07722, circRNA_02253, circRNA_06688, circRNA_04873, circRNA_04670, circRNA_04804, circRNA_ 05413, circRNA_06881, and circRNA_07666 on agarose gel electrophoresis.

To verify the accuracy of the RNA-seq data, we randomly selected nine DE circRNAs for qRT-PCR analysis: circRNA_07722, circRNA_02253, circRNA_06688, circRNA_04873, circRNA_04670, circRNA_04804, circRNA_05413, circRNA_06881, and circRNA_07666 (Figure 5 and Figure 6). The qRT-PCR-identified circRNA expression trends were consistent with the RNA-seq data (Figure 5B). The Sanger sequencing results were also consistent with the RNA-seq results (Figure 5C). PCR amplification confirmed the presence of the selected circRNAs (Figure 6). These results confirmed the authenticity of the circRNAs and reliability of the RNA-seq data.

### 3.5. Screening of DE circRNAs Related to Secondary Hair Follicle Development

We screened 16 DE circRNAs related to SF development to construct a circRNA–miRNA–mRNA interaction network. The 16 circRNAs were circRNA_07722, circRNA_00369, circRNA_01130, circRNA_15222, circRNA_08249, circRNA_00618, circRNA_04007, circRNA_01256, circRNA_02438, circRNA_12843, circRNA_13884, circRNA_05165, circRNA_05413, circRNA_04670, circRNA_04873, and circRNA_07666 (Table 2).

### 3.6. Analysis of circRNA-miRNA-mRNA Interaction Network

**Figure 7:**
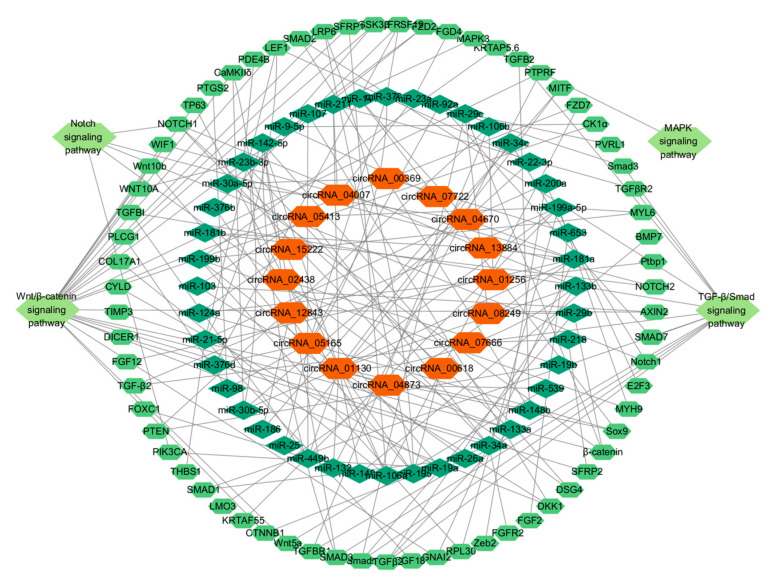
The circRNA–miRNA–mRNA regulatory network related to SF genesis and development. From the inner circle to the outer circle of circRNA–miRNA–mRNA are DE circRNAs, miRNAs, mRNAs, and signalling pathways.

To investigate the regulation of targeting and post-transcriptional effects of circRNAs on miRNAs in the skin of alpine fine-wool sheep, we used miRanda (v3.3a) and TargetScan (v7.0) to predict their interactions (Table S5). Based on the 16 differentially expressed circRNAs, 44 targeted miRNAs, and 65 associated mRNAs, 88 circRNA–miRNA–mRNA regulatory networks related to SF development were constructed (Figure 7, Table S6). The analysis revealed that multiple circRNAs synergistically regulated the same miRNAs, such as circRNA_00369, circRNA_04670, and circRNA_01130, all of which targeted miR-30a-5p.

### 3.7. Analysis of the Expression Levels of circRNAs

**Figure 8:**
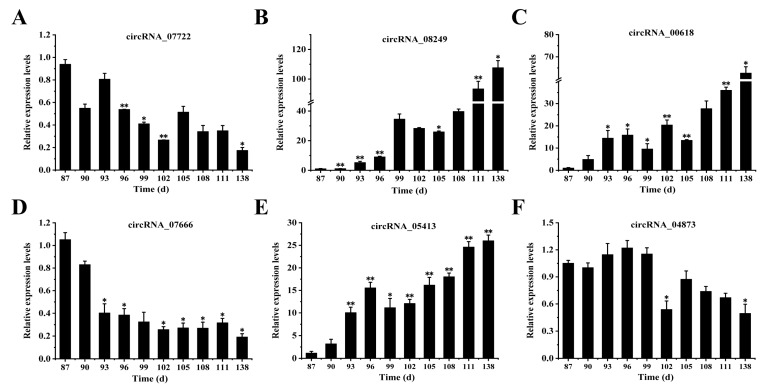
Expression patterns of circRNA. (**A**–**F**) Expression levels of six circRNAs (circRNA_07722, circRNA_08249, circRNA_00618, circRNA_07666, circRNA_05413, and circRNA_04873) related to SF development at different stages. * *p* < 0.05; ** *p* < 0.01.

We randomly selected 6 circRNAs (circRNA_07722, circRNA_08249, circRNA_00618, circRNA_07666, circRNA_05413, and circRNA_04873) from 16 circRNAs associated with the occurrence of SFs in fine-wool sheep. Foetal skin was examined at 10 different developmental stages (E87, E90, E93, E96, E99, E102, E105, E108, E111, and E138) to understand the role of circRNAs in the foetal skin of fine-wool sheep (Figure 8). The results indicated that the overall trends of the expression levels of circRNA_07722, circRNA_07666, and circRNA_04873 gradually decreased as the number of foetal days increased (Figure 8A,D,F). However, the overall trends of the expression levels of circRNA_08249, circRNA_00618, and circRNA_05413 gradually increased with increasing foetal days and reached a maximum value at 138d (Figure 8B,C,E). These findings suggested that the above six circRNAs may have potential regulatory functions in SF development in the foetal skin of fine-wool sheep through the ceRNA network.

## 4. Discussion

The growth and development of SFs in the embryonic stage as a regenerative system involves a variety of intricate molecular regulatory processes, including the genesis, proliferation, differentiation, and apoptosis of a wide range of cells [34]. Systematic research on the down production process and SF development mechanisms at different embryonic developmental stages in fine-wool sheep is lacking. Existing studies have demonstrated that the SFs of alpine fine-wool sheep originate at a gestational age of 87 days, the cervical protuberances of the primary SFs protrude and redifferentiate at 102 days, the primary SFs redifferentiate extensively at 108 days, and most of the primary SFs and some others redifferentiate at 138 days [23]. Based on this, we used RNA-seq technology to screen ceRNAs and constructed a circRNA–miRNA–mRNA regulatory network related to the occurrence and development of SFs in fine-wool sheep.

In this study, we analysed the expression characteristics of circRNAs in the HFs of fine-wool sheep at different developmental stages using RNA-seq and identified 173 DE circRNAs. Some circRNA-targeted genes have been reported to be involved in HF growth and may be important targets for research [35]. In addition, the expression profiles of circRNAs were consistent with those of their source genes [36,37]. KEGG is a pathway database for the systematic analysis of gene functions. In this study, KEGG pathway analysis revealed that Wnt, TGF-β, Notch, NF-κB, PI3K-Akt, P53, and AMPK were significantly involved in SFs (*p* < 0.05). Based on the significant KEGG pathways, 16 circRNAs were initially screened (circRNA_07722, circRNA_00369, circRNA_01130, circRNA_15222, circRNA_08249, circRNA_00618, circRNA_04007, circRNA_01256, circRNA_02438, circRNA_12843, circRNA_13884, circRNA_05165, circRNA_05413, circRNA_04670, circRNA_04873, and circRNA_07666). The genes from which these circRNAs originate (*COL15A1*, *DOCK7*, *ADAMTS5*, *HADH*, *COL3A1*, *TGFBR3*, *ISM1*, *ARL13B*, *POSTN*, *KCTD9*, *KRT77*, *SEMA3C*, *CTNND1*, *DKK3*, *ZNF114*, and *FTO*) are involved in the HF growth process [38,39,40,41,42,43,44,45,46,47,48,49,50,51,52]. A comparison of our results with those of previous studies suggests that the selected circRNAs may play important roles in signalling pathways at different stages of SF development in fine-wool sheep. Further studies are required to determine the exact mechanisms involved.

According to the ceRNA hypothesis, circRNAs regulate the expression of target genes by competitively binding to miRNAs [53]. In this study, 44 circRNA-targeting miRNAs associated with the development of SFs were predicted using miRanda (v3.3a) and TargetScan (v7.0) software. The Wnt/β–catenin [54], TGF-β/Smad [55], Notch [56], and MAPK [57] signalling pathways are essential in HF growth. The Wnt signalling pathway plays a central role in the regulation of follicle formation and cycle regeneration induced by HF papilla cells [58]. In the TGF-β signalling pathway, TGF-β1 knockdown delays the transition of the HF from the anagen to the anagen phase [59]. TGF-β2 inhibits the elongation of the hair shaft and induces changes in the morphology of the HF during the anagen phase of the HF [60]. The Notch signalling pathway is activated under the regulation of Tβ4, which in turn drives the proliferation, differentiation, and migration of HF cells [61]. The MAPK signalling pathway is essential for the regulation of mammalian cell proliferation [56].

Recent studies revealed that some circRNAs contain multiple miRNA binding sites [18,53]. As circRNAs cannot directly regulate their target genes, they act as ‘miRNA sponges’. CircRNAs are involved in many biological processes by acting as miRNA sponges, thus eliminating the inhibitory effects of miRNAs on their target genes [53]. CircRNAs can regulate gene expression through the circRNA–miRNA–mRNA pathway [36]. However, the dynamic changes and functions of circRNAs at different developmental stages in fine-wool sheep SFs remain understudied. CircZNF423, a ceRNA that regulates the expression of oar-miR-541-3p and targets *CALM3*, inhibits the proliferation of sheep myoblasts [62]. In Hoop, circFTO and circCSPP1 competitively bind to miR-148a and miR-10a to deregulate the inhibitory effect of miRNAs on *BMP7*, which in turn promotes *BMP7* expression and drives the proliferation of sheep wool cells [63]. Furthermore, circRNA-1967 upregulates the expression of *LEF1* by adsorbing miR-93-3p, which is involved in HF-related regulation of cellular function [64]. In the hair papilla sheaths of velvet goat SFs, circRNA-1926 was shown to target and regulate cell cycle protein-dependent kinase 19 (*CDK19*) gene expression and mediate HF stem cell differentiation into SHFs via miR-148a/b-3p [64]. These findings suggest that circRNAs competitively bind miRNAs through the ceRNA mechanism to regulate the expression of key genes, which in turn affect HF growth and differentiation.

Based on the results of KEGG pathway analysis combined with DE circRNA data related to the occurrence of SFs in fine-wool sheep, we successfully constructed a circRNA–miRNA–mRNA interaction network containing 16 circRNAs, 44 miRNAs, and 65 mRNAs. This network provides important clues for the in-depth characterisation of the SF in sheep at different developmental stages. Previous studies have successfully predicted 102 pairs of circRNA–miRNA interactions and 126 pairs of miRNA–mRNA interactions [65], confirming that there is a targeted regulatory relationship between circRNAs and specific miRNAs, and that circRNAs can indirectly regulate target gene expression through miRNAs. In this study, 16 screened circRNAs were integrated and analysed with the predicted miRNA and mRNA sequence data, and 88 sets of circRNA–miRNA–mRNA interactions were identified. We hypothesised that the newly identified circRNAs might be involved in regulating the growth and development of HFs in fine-wool sheep through similar molecular mechanisms. In addition, the expression levels of six circRNAs (circRNA_07722, circRNA_08249, circRNA_00618, circRNA_07666, circRNA_05413, and circRNA_04873) were examined in this study at 10 different developmental stages of wool follicles in sheep embryos. The results showed that the expression of these circRNAs changed dynamically with the developmental stage. Future studies should focus on the specific mechanisms underlying the roles of the candidate circRNAs (circRNA_07722, circRNA_08249, circRNA_00618, circRNA_07666, circRNA_05413, and circRNA_04873) in HF development.

## 5. Conclusions

In this study, we constructed the expression profiles of circRNAs in the HFs of fine-wool sheep at different embryonic stages (E87, E90, E93, E96, E99, E102, E105, E108, E111, and E138), and a total of 173 DE circRNAs were identified. The results of GO and KEGG analyses suggest that there may be a relationship between circRNAs and their target genes. CircRNAs were found to play a role in HF growth and development in fine-wool sheep through signalling pathways such as Wnt/β-catenin, TGF-β/Smad, Notch, and MAPK. We examined the expression levels of six circRNAs associated with HF development at different embryonic stages that may regulate SF development in fine-wool sheep through the ceRNA network. A circRNA–miRNA–mRNA regulatory network was also constructed, and 88 interacting miRNA–mRNA pairs were investigated. The candidate circRNAs, including circRNA_07722, circRNA_08249, circRNA_00618, circRNA_07666, circRNA_05413, and circRNA_04873, provide targets for future studies on wool follicle regulation.

## Figures and Tables

**Table 1:** Information on qRT-PCR primer sequences.

circRNAs	Forward Primer Sequence	Reverse Primer Sequence
circRNA_07722	AGCCTTCCAATAGTGAACCTCA	GCAACCAAGTGTAGTGCAGG
circRNA_02253	TGAAACACAGTTGGCAGAGTCT	ACGGCAAGGCACCAAACT
circRNA_06688	TGGTGAAGACATTGCAGACGA	AGCTCATTCACTTGTTCTCTCCA
circRNA_04873	GCGGATGAAAGGAATGGGGA	TGCTCTGTTGACTCCCTGAAA
circRNA_06881	GAGAACCAGTGGCTGCGG	GCTTCTCCAACTTGTCCTCCT
circRNA_04670	TCGAGTTTGAATGGCTGAGACA	AGATGTATTCCAAGGTCCCCG
circRNA_04804	GTTGGAGCAGGAAGAGGAGC	TGAGATAGCAGGAGTTTGGAAGAC
circRNA_07666	TTTCTCGATTGGACCTGCGA	GGGTGAATGATCCTCTGGTGG
circRNA_05413	GGAGATCCCGCAAGCAGAG	AGCTTGCTATCCGAGTCTTTCT
circRNA_03494	ATTGTTGCCTACGCCCACTT	GCCATTCGTGAACAGCATCG
circRNA_08249	AAGGGTGATTCTGGTGCTCC	CAGACCAGGTGTACCAGCAG
*β*-actin	CAGTCGGTTGGATGGAGCAT	AGGCAGGGACTTCCTGTAAC

**Table 2:** Statistics of 16 circRNAs’ information related to the development of secondary HFs.

circRNA ID	Source Gene	*p*-Value	Up/ Down	circRNA Size	Isoform Name
circRNA_07722	COL15A1	2.29 × 10^−2^	Down	362	XM_027964395.1
circRNA_00369	DOCK7	7.36 × 10^−3^	Down	429	XM_015092012.2
circRNA_01130	ADAMTS5	2.03 × 10^−3^	Down	301	XM_015092381.2
circRNA_15222	HADH	4.82 × 10^−2^	Down	504	XM_004009637.4
circRNA_08249	COL3A1	7.73 × 10^−3^	Up	315	XM_004004514.4
circRNA_00618	TGFBR3	4.99 × 10^−2^	Up	871	XM_012176015.3
circRNA_04007	ISM1	2.15 × 10^−2^	Up	739	XM_027976478.1
circRNA_01256	ARL13B	1.43 × 10^−2^	Up	1077	XM_004002849.4
circRNA_02438	POSTN	4.29 × 10^−2^	Down	570	XM_004012110.4
circRNA_07666	KCTD9	3.92 × 10^−2^	Down	314	XM_027964318.1
circRNA_12843	KRT77	8.74 × 10^−4^	Up	12,347	XM_027967277.1
circRNA_13884	SEMA3C	1.06 × 10^−2^	Down	1027	XM_004007807.4
circRNA_05413	CTNND1	2.03 × 10^−2^	Up	272	XM_027979807.1
circRNA_05165	DKK3	2.54 × 10^−2^	Down	222	XM_027979387.1
circRNA_04873	ZNF114	4.36 × 10^−2^	Down	1359	XM_027978554.1
circRNA_04670	FTO	4.35 × 10^−2^	Down	344	XM_027977360.1

## Data Availability

Data are available upon request due to privacy/ethical restrictions.

## Supplementary Materials

The following supporting information can be downloaded from https://www.mdpi.com/article/10.3390/ani15172629/s1: Table S1: Summary table of sequencing data screening; Table S2: Differentially expressed circRNAs (DE circRNAs) among different groups; Table S3: KEGG enrichment results of DE circRNA source genes; Table S4: GO enrichment results of DE circRNA source genes; Table S5: All predicted target miRNAs of DE circRNAs; Table S6. Construction of DE circRNA–miRNA–mRNA interaction network.
